# Effects of double vs triple injection on block dynamics for ultrasound-guided intertruncal approach to the supraclavicular brachial plexus block in patients undergoing upper limb arteriovenous access surgery: study protocol for a double-blinded, randomized controlled trial

**DOI:** 10.1186/s13063-022-06260-6

**Published:** 2022-04-12

**Authors:** Quehua Luo, Huiying Liu, Longjiao Deng, Lidan Nong, Haifeng Li, Yujing Cai, Junyi Zheng, Haihua Shu, Weifeng Yao, Jianxing Zhang

**Affiliations:** 1 Department of Anesthesiology, Guangdong Provincial People’s Hospital, and Guangdong Academy of Medical Sciences, Guangzhou, Guangdong 510080 People’s Republic of China; 2 Department of Anesthesiology, the First Affiliated Hospital of Guangzhou University of Chinese Medicine, Guangzhou, 510405 Guangdong China; 3 Department of Anesthesiology, the Third Affiliated Hospital of Sun Yat-Sen University, Guangzhou, 510630 Guangdong China

**Keywords:** Intertruncal approach, Ultrasound, Brachial plexus, Supraclavicular block, Injection technique

## Abstract

**Background:**

Ultrasound-guided intertruncal approach (IA) has been proposed to be an alternative and promising approach to the supraclavicular block (SCB), in which double injection (DI) of local anesthetics (LA) is sequentially administered between intertruncal planes. We would like to apply a refined injection technique, named triple injection (TI) technique, based on the 3 separate compartments visualized by ultrasound. The aim of this study is to compare the percentage of patients with complete sensory blockade at 20 min of DI vs TI technique, when they are applied in patients undergoing upper limb arteriovenous access surgery.

**Methods:**

This study is a prospective parallel-group randomized controlled trial. A total of 86 end-stage renal disease patients will be randomly allocated to receive IA-SCB using either DI or TI technique with identical LA (0.5% ropivacaine 24 mL). The primary outcome is the percentage of patients with complete sensory blockade of all 4 terminal nerves (median, ulnar, radial, and musculocutaneous nerves) of the brachial plexus measured at 20 min after injection. The secondary outcomes will consist of the sensory or motor blockade of all individual nerves, onset times, performance time, diaphragmatic paralysis, surgical anesthesia, and adverse events.

**Discussion:**

It is expected that ultrasound-guided IA-SCB with the TI technique results in better block dynamic in patients undergoing upper limb arteriovenous access surgery.

**Trial registration:**

Chinese Clinical Trial Registry ChiCTR2100045075.

## Introduction

Supraclavicular block is the most common and preferred anesthesia method for arteriovenous (AV) fistula formation surgery [1]. Since nerve blocks tend to increase vasodilation, blood flow, and tissue oxygenation during autogenous AV fistula creation, they improve the successful rate of fistula [2]. Furthermore, supraclavicular block anesthetizes the brachial plexus at the level of the first rib, where all trunks and divisions are in their most compact form, thus providing a complete and reliable block for upper extremity surgery [3]. Although various ultrasound-guided needle techniques to supraclavicular block have been reported to have satisfying success rate and onset times, these techniques have been associated with an incomplete blockade for the ulnar nerve innervated area and a high risk of intraneural injection, respectively [3, 4]. The intertruncal approach to the supraclavicular brachial plexus (IA-SCB) was first described by Siddiqui et al. [5] in 2020, in which the needle tip targets the two intertruncal tissue planes between the 3 trunks. In authors’ experience, this new approach results in rapid and consistent blocking of all terminal nerves of the brachial plexus. Compared with the sub-epineurium techniques to the supraclavicular block, its superiority is to provide complete sensory-motor blockade of the entire brachial plexus while avoiding intraneural injection and pleural puncture on anatomy.

However, the biggest challenge of targeting two intertruncal planes is that we do not know exactly where our needle tip lies between the two planes. It may lie in an incorrect location, even as we see the needle tip in place on ultrasound imaging. Because the homogeneous fascia tightly compresses together, it does not allow for easy identification of these elements as separate structures [5]. Incorrect injection probably results in an atypical diffusion pattern of LA and following incomplete block. In clinical practice, we observe that the 3 trunks can be separated by clear edges rather than being a singular cluster, the outer boundaries (epineurium) of each trunk allow injection of LA into the 3 separate compartments under real-time ultrasound guidance. Therefore, we refined the needle technique by depositing LA inside the 3 trunks separately (named triple injection, TI) with clear identification of the epineurium of each trunk, that is the needle tip will be accurately pierced into the epineurium in a “bottom to top” manner.

Needle techniques for traditional supraclavicular blocks include sub-epineurium injection (e.g., double injection [6]), intracluster injection (e.g., triple [7], targeted intracluster injection [8, 9]), or extra-epineurium injection (e.g., corner pocket injection [10]). Although the total anesthesia-related times vary in those studies, the results have demonstrated that the multiple injection technique is feasible and can achieve faster onset times and higher success rates within 30 min after injection, with no increase in complications [11]. The triple-injection technique described by Arab et al. [7] was designed based on the clusters of the brachial plexus at the level of the first rib, which can be artificially divided into three parts. The percentage of patients with complete sensory blockade was up to 76% in TI vs 56% in single injection for upper limb arteriovenous access surgery at 20 min after injection. However, a cadaver study has reported that even single intracluster injection could lead to a high risk of sub-perineurium injections (up to 24%) in the traditional supraclavicular block, which raised concerns about nerve injury [4]. Fortunately, the 3 separate compartments visualized during the intertruncal approach are naturally occurring along the trajectory of the brachial plexus, in which each trunk is separated not yet blended. Consequently, in this trial, we will compare DI vs TI ultrasound-guided IA-SCB. Based on our initial experience, we hypothesized that the new TI technique will result in a higher percentage of patients with complete sensory blockade in the first 20 min, while avoiding puncture-related adverse events.

## Methods and analysis

### Trial design

**Fig. 1:**
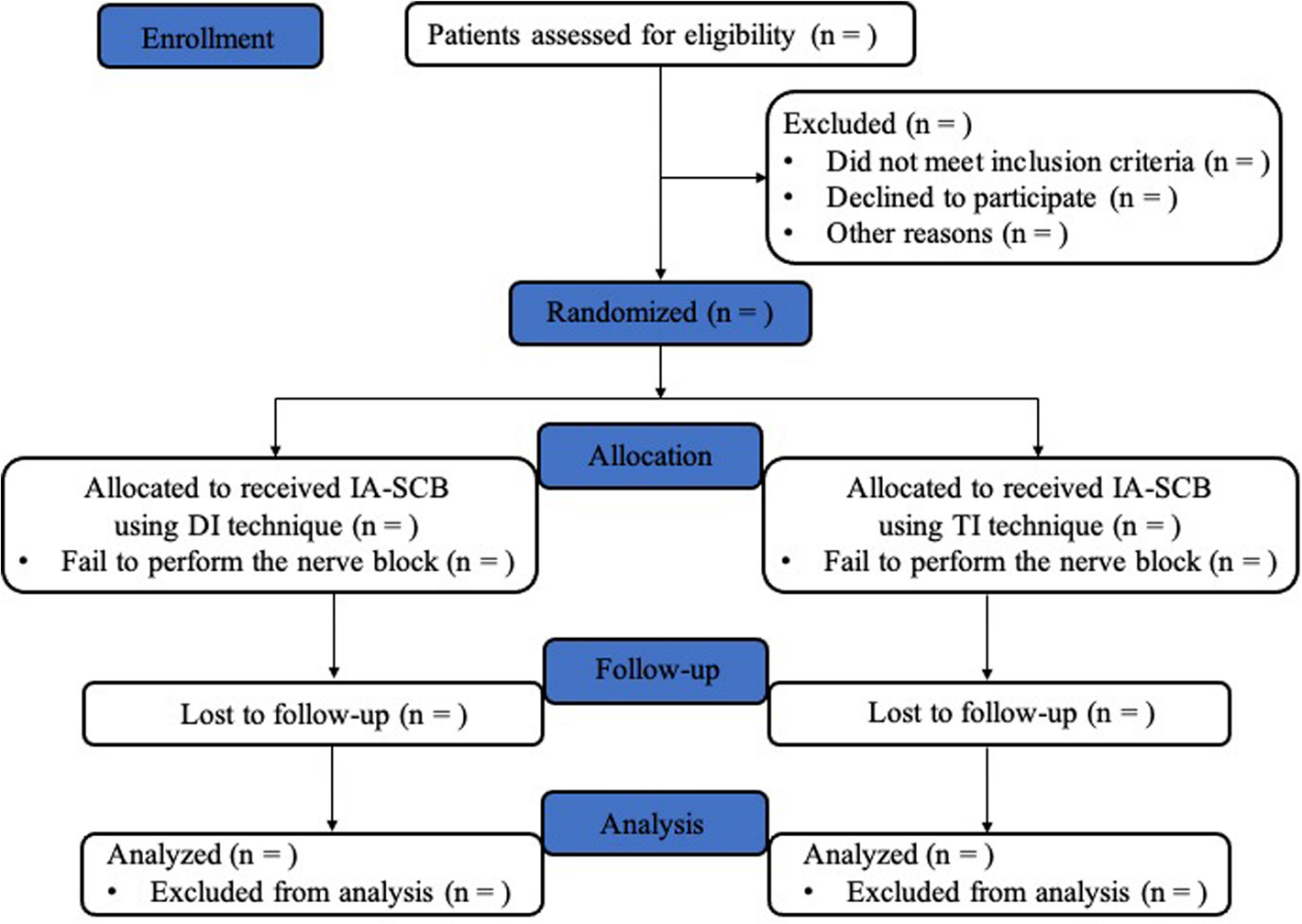
Flow diagram of this study according to Consolidated Standards of Reporting Trials (CONSORT) 2010 guidelines. IA, intertruncal approach; SCB, supraclavicular block; DI, double injection; TI, triple injection

**Fig. 2:**
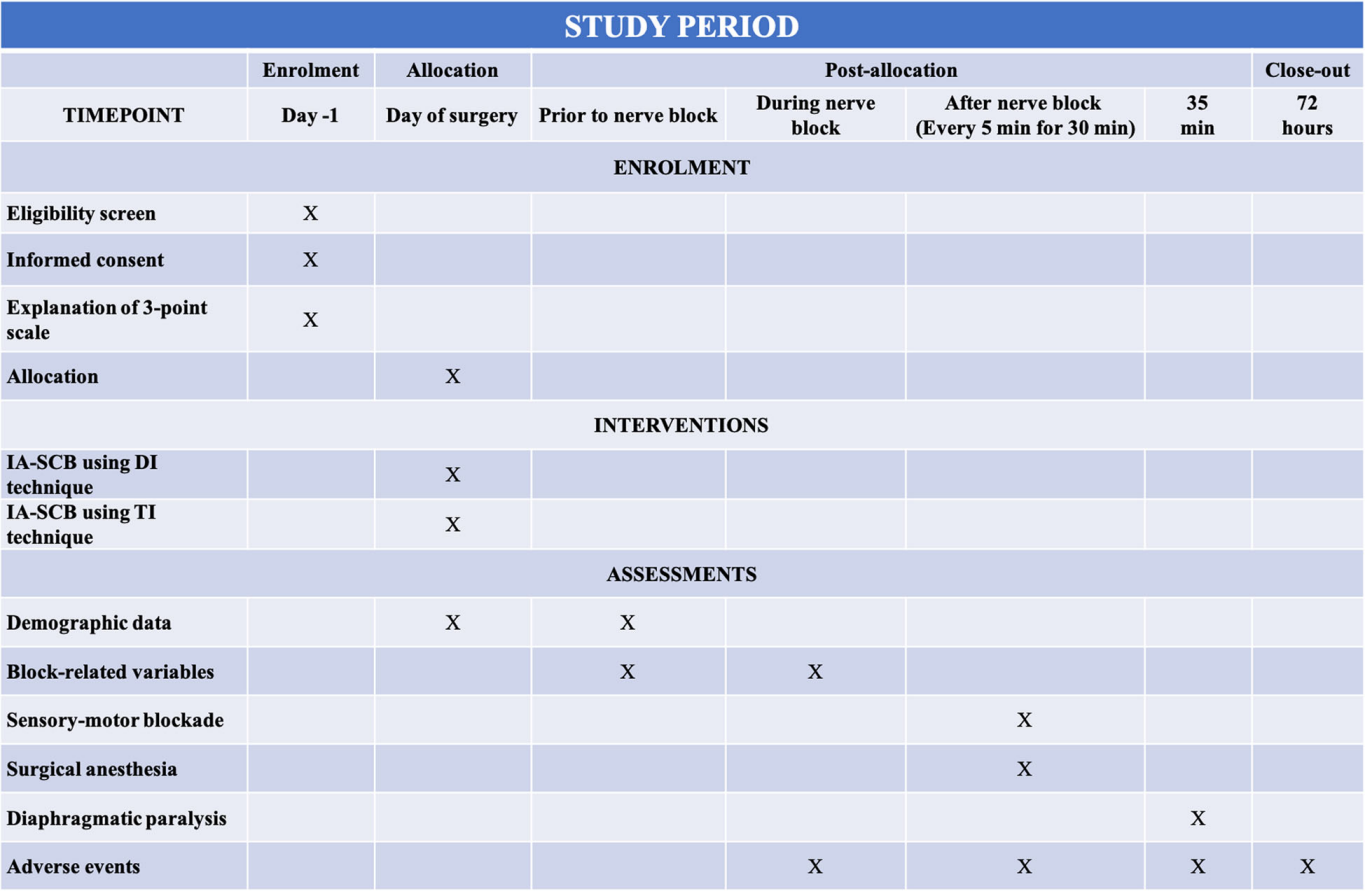
Standard Protocol Items: Recommendations for Interventional Trials. IA, intertruncal approach; SCB, supraclavicular block; DI, double injection; TI, triple injection

This study is a single-center, randomized, double-blind, parallel-group clinical trial with concealed 1:1 allocation to receive either a DI or TI technique to IA-SCB of patients scheduled for elective upper limb arteriovenous access surgery. The trial will be carried out at the Guangdong Provincial People’s Hospital, Guangzhou, Guangdong, China, and started in July 2021 and the recruiting period will be 18 months. Trial methods and results will be reported according to the Consolidated Standards of Reporting Trials (CONSORT) 2010 guidelines (Fig. 1). The schedule of enrolment and assessments is shown in the Standard Protocol Items: Recommendations for Interventional Trials (Fig. 2).

### Participant eligibility and consent

The investigators involved in this trial will identify consecutive eligible patients according to the listed criteria. The benefits, risks, and data privacy of this study will be explained in detail during the preoperative visit 1 day before surgery. Written informed consent will be obtained from each patient before enrollment. This study is voluntary, and the participants are free to withdraw from the study at any time.

### Inclusion criteria

- Having signed the written informed consent
- American Society of Anesthesiologists (ASA) physical status < IV
- Aged 18–75 years
- Patients scheduled for creation or superficialization of arteriovenous fistula or aneurysmectomy or arteriovenous fistula with artificial vessels for renal dialysis

### Exclusion criteria

- Patient’s refusal to brachial plexus block
- Coagulopathy: INR ≥ 1.4, or PLT < 100 × 10^9^/L, or APTT ≥ 40s, or PT ≥ 17 s
- Pre-existing neuropathy
- Infection at the supraclavicular fossa
- Hypersensitivity or allergy to LA
- Severe pulmonary disease or poor respiratory reserve: patients require home oxygen therapy or non-invasive ventilation, or limit exercise tolerance to < 4 METs (maximum exercise tolerance)
- Severe hepatic insufficiency: Child-Turcotte-Pugh score > 9
- Severe cardiac insufficiency: cardiac conditions that limit exercise tolerance to < 4 METs or left ventricular ejection fraction (LVEF) < 40%
- Severe mental illness or cognitive dysfunction
- Body mass index (BMI) > 35 kg/m^2^
- Pregnancy or breastfeeding women
- Urgent surgery

### Dropout criteria

- Patients voluntarily withdraw from the study
- Change of operation or anesthesia methods before or during surgery
- Patients do not cooperate with postoperative follow-up
- Not implemented according to the study plan

### Allocation and blinding

- Sequence generation: Randomization will be performed based on 9 permuted blocks with block sizes of 10 for covering the total sample size. This will be done using computer-generated random numbers (Randomization.com).
- Allocation concealment mechanism: The investigator (JX.Z. and WF.Y.) will be responsible for patient recruitment. Randomization will be done by a settled research assistant during the study period. A sealed opaque envelope containing a card with a randomization number generated by a computer will be opened on the day of surgery.
- Implementation: The nerve block will be carried out by one of the five investigators (QH.L., LJ.D., LD.N., YJ.C., and HF.L.) according to treatment allocation, who all have extensive experience with both techniques (over 60 attempts/per technique) before the study
- Blinding (masking): Outcome assessments will be conducted by one blinded anesthesiologist of assessment staff who will not communicate with the nerve block operators. During the study period, patients, outcome evaluator, and follow-up personnel (two research assistants) will be kept blinded to the group allocation. If block-related serious adverse events (e.g., pneumothorax or LA systemic toxicity) occur during or after the nerve block procedure, un-blinding will be permissible, and the corresponding treatment measures will be initiated under the supervision of the chief investigators (JX.Z. and WF.Y.).

### Interventions

All the enrolled patients will be randomly allocated to one of the following two study groups:

- Control group: patients will receive a standardized IA-SCB using the DI technique.
- Experimental group: patients will receive a modified IA-SCB using the TI technique.

### Ultrasound-guided nerve block techniques

Before starting the study, all included patients will be instructed on the use of a validated 3-point scale for evaluating sensory-motor blockade. None of the patients will receive premedication. Standard ASA monitoring, including non-invasive cuff blood pressure, pulse oximetry, and 5-lead electrocardiogram, will be applied in the operating room. An intravenous access (18- or 20-gauge) will be established in the contralateral forearm, and the premedication (midazolam 1–3 mg and fentanyl 0.5 μg/kg) will be administered as anxiolytic. Supplemental oxygen (total flow, 4 L/min) will be given via nasal cannula during nerve block and surgery. For ultrasound-guided IA-SCB, the nerve block will be performed with ultrasound (Philips CX50) and high-frequency (3–12 MHz) linear array probe and 80-mm short-beveled stimulating needle (B. Braun Melsungen AG, Melsungen, Germany) following standard skin disinfection.

**Fig. 3:**
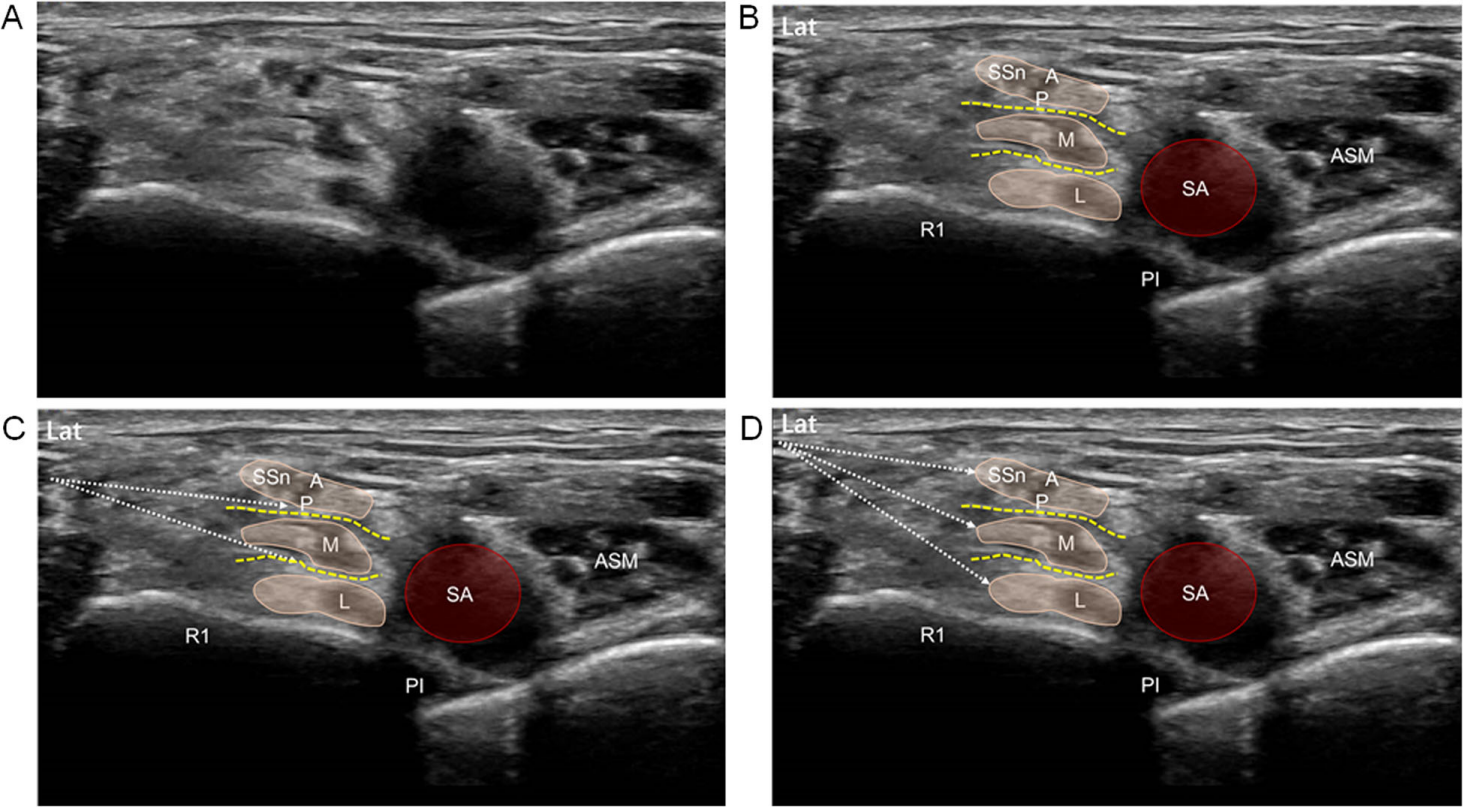
Images demonstrating the satisfactory imaging for IA-SCB (**A**, **B**) and the double-injection (**C**) and triple-injection (**D**) techniques used in IA-SCB. **A**, **B** The satisfactory imaging of the individual trunks of the brachial plexus in the supraclavicular fossa. **C** Sites of injection in the DI method: the needle tip (dotted arrows) is orientated to the intertruncal planes of the 3 trunks (first injection in the lower intertruncal plane, second in the upper plane), and the desired spread pattern of LA will be confirmed by ultrasound. **D** Sites of injection in the TI method: the needle tip (dotted arrows) is orientated orderly to the outer boundaries (epineurium) of the trunks, and the desired spread pattern of LA will be confirmed by ultrasound. The injection sequence is from bottom to top, that is the first injection for the lower trunk, second for the middle trunk, and last for the upper trunk. A, anterior division of upper trunk; p, posterior division of upper trunk; SSn, suprascapular nerve; L, lower trunk; M, middle trunk; Pl, pleura; R1, first rib; SA, subclavian artery; dotted line = intertruncal planes; ASM, anterior scalene muscle; LA, local anesthetics; IA, intertruncal approach; SCB, supraclavicular block; DI, double injection; TI, triple injection

The DI technique will be performed according to the method described by Siddiqui et al. [5], and an optimal order of injections will follow the suggestion in Endersby’s Letter [12]. The probe is initially placed in the supraclavicular fossa and then moves towards the base of the neck in a coronal oblique plane. To obtain a satisfactory image, the probe will be adjusted with a slight posterior tilt and moved side to side. The 3 trunks (upper, middle, and lower) with individual outer boundaries (epineurium) are clearly identified in the short axis. In this area, all 3 trunks are displayed as three fascial compartments lateral to the subclavian artery, in which the upper trunk is the most superficial, the lower trunk is located above the first rib, and the middle trunk is just sandwiched between two trunks (Fig. 3A, B). After obtaining a satisfactory image, we used a pre-injection technique with 3 mL of LA for the superior identification of epineurium before administering the first LA dose. For the DI technique, the operators initially orientate the needle tip to the lower intertruncal tissue plane (between the middle and lower trunks), and a total of 12 mL of 0.5% ropivacaine (Astrazeneca Pharmaceutical Co., Ltd.) will be injected. If the first 1–2 mL LA does not demonstrate optimal spread between two planes, the needle tip will be repositioned slightly in “advance or retreat” manners until the desired spread pattern of LA is visualized. Subsequently, the needle withdraws and targets the upper intertruncal tissue plane (between the upper and middle trunks) (Fig. 3C). The remaining volume (12 mL) will be carefully injected incrementally into that plane. For the TI technique, once a satisfactory image is obtained, the operators accurately orientated the needle tip into the epineurium of the 3 trunks in a “bottom to top” manner using an in-plane technique. That is, LA is injected in 3 aliquots of 8 mL each. Aliquots will be deposited sub-epineurium around the upper, middle, and lower trunks respectively (Fig. 3D).

### Outcome measures

#### Primary outcome measure

The primary outcome will be the percentage of patients with complete sensory blockade of all 4 terminal nerves (median, ulnar, radial, and musculocutaneous nerves) of the brachial plexus measured at 20 min after injection. The sensory blockade will be evaluated using a validated 3-point scale (0 = no block, the patient has normal sensation; 1 = partial anesthesia, the sensation is reduced compared with the contralateral limb; and 2 = complete anesthesia) by an outcome evaluator (either anesthesiologist or anesthesia nurse). Sensory blockade of the musculocutaneous, median, radial, and ulnar nerves will be evaluated on the lateral aspect of the forearm, the volar aspect of the thumb, the lateral aspect of the dorsum of the hand, and the volar aspect of the fifth finger, respectively. The complete sensory for each innervated nerve was defined as score is equal to 2 points. We will consider the patients having a complete sensory blockade when a minimal composite score of 7 points is achieved.

#### Secondary outcome measures

- The onset time is defined as when a minimal composite sensory-motor score of 14 points is achieved. The assessment of sensory-motor blockade will be performed every 5 min until 30 min after injection. The above 3-point scale is used for evaluating sensory blockade, and another similar 3-point scale (0 = no block, 1 = paresis, 2 = paralysis) for motor blockade. Motor blockade of the musculocutaneous, radial, median, and ulnar nerves will be evaluated by elbow flexion, thumb abduction, thumb opposition, and thumb adduction, respectively.
- The performance time (including imaging time and needle time) is defined as the time interval between contact of the ultrasound probe with the skin and complete injection of LA.
- The incidence of diaphragmatic paralysis measured with M-mode as described by Boussuges et al. [13] at 35 min after injection.
- Successful surgical anesthesia is defined as one with adequate surgical block without complaining pain during simulated surgical stimulus (forceps pinching the prospective surgical area) at 30 min after injection.
- Operation-related adverse events include puncture-related paresthesia, procedural-related pain, Horner syndrome, vascular puncture, pneumothorax, toxicity of LA, or neurological deficits. The puncture-related paresthesia will be recorded by self-report during performing nerve block, and the procedural-related pain is assessed by the numeric rating scale (0 = no pain, 10 = worst possible pain). Horner syndrome, vascular puncture, pneumothorax, and toxicity of LA will be recorded as the incidence in each group. All patients will be followed up for 72 h after surgery for suspicious symptoms of nerve injury such as paresthesia or motor deficit.

### Sample size calculation

Sample size is calculated according to the study of Arab and colleagues [7]. In clinical practice, multipoint injection and satisfactory diffusion of LA will result in more complete sensory or motor blockade and faster onset times. Therefore, we expected a higher rate of complete sensory blockade using the new TI technique. Based on a pilot study (15 patients), the rate was 66.7% (patients with complete sensory blockade) at 20 min after injection, when patients received a standardized IA-SCB with the DI technique. We hypothesized that the TI technique will be capable of increasing this rate to 90%; with a statistical power of 0.80 and a *α* level of 0.05, the required sample size is calculated to 36 patients per group. A total of 43 patients per group will be required to account non-included patients (15% dropout rate).

### Statistical plan

Statistical analysis will be performed using SPSS version 20 statistical software (SPSS Inc, Chicago, IL). Firstly, an overall descriptive analysis and inferential statistics for all clinical variables in both groups will be performed. Numbers and percentages will be recorded for categorical variables, and mean (SD) and interquartile intervals for continuous variables. For all data, normal distribution will be examined by the Kolmogorov-Smirnov test. The basic information (such as age, BMI, and performance times, etc.) will be analyzed using chi-square tests or Fisher’s exact test or independent *t* test as appropriate. The percentage of patients with a complete sensory blockade at all predetermined time points and onset times will be analyzed using the Mann-Whitney *U* test or independent *t* test. The incidence of diaphragmatic paralysis, surgical anesthesia, and operation-related adverse events will be analyzed using chi-square tests or Fisher’s exact test. A *P* value inferior to 0.05 will be considered statistically significant for all results.

### Reporting of adverse events

All block-related adverse events will be closely monitored and recorded in the “adverse events form,” in which the detail consists of the time of occurrence, diagnose or suspect diagnose, severity and intensity, relationship with the nerve block, treatment measures, and outcome. All adverse events will be dealt with the formulated treatment measures until a stable situation is reached. If a serious adverse event persists at the end of the study, the investigator must follow the patient until the event is considered resolved. The form will be checked and analyzed at the predetermined time of interim analyses. The chief investigator will be informed of any serious adverse events (e.g., pneumothorax, toxicity of LA, or neurological deficits) and report these to the Institutional Review Board immediately.

### Ancillary and post-trial care

At the end of the study, patients in both groups will have an option to be evaluated for recovery of sensory-motor functions of the brachial plexus to compensate at the anesthesia clinic 7 days after surgery.

### Data collection and retention

All recorded variables will be desensitized and stored securely at the Department of Anesthesiology of Guangdong Provincial People’s Hospital for 5 years. Preserved paper materials consist of the original signed informed consents, study protocol and interventions, and case report form in this study. These data will not be revealed to other people without appropriate permission.

### Patient and public involvement

None of the patients and public will be involved in the design, recruitment, and dissemination plans of the study. Three or four patients will participate in the study voluntarily prior to including 43 candidates (half of the total sample size). They will provide their own experience on the sensorimotor blockade and performing the nerve block during this study, also including their most troubling problems and elements of this study that need to be improved. At the end of the study, an additional three or four patients will be interviewed to record the improvement of the above problems in preparation for further study of this technique.

### Protocol amendments

In principle, the established study protocol is not allowed to be modified, except for safety concerns. Any amendments will be first proposed by the principal investigators and then agreed and confirmed by all co-authors. Finally, the modified version of the protocol will be submitted to the Institutional Review Board for approval.

### Ethics and dissemination

#### Ethical and legislative approvals

After being approved by the Institutional Review Board, this trial has been registered in the China Clinical Trial Registry on April 6, 2021 (identification number: ChiCTR2100045075). Any revision or modification in the approved protocol will be notified to the Ethics Committee for approval.

### Trial dissemination

The research results or findings will be disseminated in a peer-reviewed journal and at scientific conferences.

## Discussion

This is a prospective, single-center, randomized, parallel-group controlled trial to assess the block dynamic (sensory-motor blockade and onset times) of DI vs TI in ultrasound-guided IA-SCB in patients undergoing upper limb arteriovenous access surgery. Based on the findings in Reina’s study [14], the LA is probably deposited between the epineurium of the trunks for the DI technique, which was described as the “investing adipose layers” in Siddiqui’s study [5]. However, the epineurium is usually reinforced by a closed paraneurium layer, which acts as an important tissue barrier to obstruct the spread of LA, thus delaying its block effects; similar findings have been reported in the costoclavicular space [15, 16]. Several studies comparing sub-epineurium vs extra-epineurium injection for brachial plexus blocks have demonstrated that deliberately penetrating the epineurium rather than the internal epineurium layer is more successful, resulting in faster onset times and higher success rates with no increased incidence in complications [11, 17]. This led our team to speculate that, if LA is injected inside the clearly outer boundaries (epineurium) of the trunks in IA-SCB respectively, it could achieve faster onset times and reliably sensory blockade of all 4 terminal nerves of the brachial plexus. Furthermore, knowing where the needle tip now, we can in good conscience advocate for an injection technique that has identical success rates but avoid needle-nerve contact as much as possible [18].

Although our study has many similarities to Arab’s study [7], some important differences are worth noting: (1) The TI is not an intracluster-injection technique, rather than a precise sub-epineurium injection technique (puncture the epineurium of the trunks under real-time ultrasound guidance); (2) compared with the classical approach to supraclavicular block, the new intertruncal approach provides clearer anatomical details, including the 3 trunks and its hyperechoic epineurium layers, which may guide the needle tip to the targeted location without intraneural injection. Furthermore, small nerves arise from the fascicles, and the intracluster-injection technique may result in sub-perineurium injections. The TI technique deposits the LA away from the trunks, even if the needle is advanced within the neurovascular sheath. Therefore, the greatest advantage of this TI technique is that keeping the needle tip outside of the perineurium as far as possible on ultrasound imaging. We also observe the puncture-related variables (paresthesia and procedural-related pain), diaphragmatic paralysis, and neurological deficits to evaluating the safety of this TI technique. Therefore, ultrasound-guided IA-SCB with the TI technique has the potential to be as “all for one” technique of the whole upper extremity similar as selective trunk block [19, 20], which can provide more complete blockade and rapid onset time with low incidence of adverse events.

This study protocol has several limitations. First, this is a single-center study and has all the limitations inherent in that study design. Second, our study will focus on the block dynamic in patients undergoing a same type of surgery, which is insufficient statistical power for rare but serious complications. The prospective or retrospective clinical study with a large sample will be desired in the future for the incidence of nerve injury and pneumothorax. Third, we cannot completely rule out that the skill level of the operators affects the block dynamic and puncture-related complications, even if we chose all experienced ones in this study protocol.

In conclusion, this trial is the first prospective, randomized, parallel, double-blind study evaluating the block characteristics for DI vs TI technique when used in new IA-SCB in patients undergoing upper limb arteriovenous access surgery. If the TI technique yields better block dynamic, it would bring strong data to promote the TI technique in IA-SCB. It may provide us with an ideal needle technique for optimized risk-benefit and advance the understanding of the optimal site of injection and optimization of the diffusion of LA in IA-SCB.

### Trial status

The study will be conducted over a period of about 18 months (from 1 July 2021 to 31 December 2022) with the latest version of the protocol. At the time of this manuscript submission, candidates had been enrolled and some patients had participated in the study.

### Audits

Throughout the entire process of this study will be monitored by a data monitoring committee (DMC) consisting of specialists in ethics, anesthesiology, and statistics in our medical center, which will audit through regular interviews or telephones. The DMC reserves the right to audit the recruitment of patients and collected data at any time, and the auditing process will be independent from the investigators.

## Data Availability

Not applicable.

## Abbreviations

IA: Intertruncal approach
SCB: Supraclavicular block
LA: Local anesthetics
DI: Double injection
TI: Triple injection
CONSORT: Consolidated Standards of Reporting Trials
ASA: American Society of Anesthesiologists
BMI: Body mass index
AV: Arteriovenous
